# Determining similarity of scientific entities in annotation datasets

**DOI:** 10.1093/database/bau123

**Published:** 2015-02-27

**Authors:** Guillermo Palma, Maria-Esther Vidal, Eric Haag, Louiqa Raschid, Andreas Thor

**Affiliations:** ^1^Departamento de Computación Universidad Simón Bolívar, Caracas, Venezuela, ^2^Department of Biology, University of Maryland, College Park, MD, 20742 USA ^3^Smith School of Business, Institute of Advanced Computer Studies, and Department of Computer Science. College Park, MD, 20742 USA and ^4^University of Applied Sciences for Telecommunications, Leipzig, Germany 04277

## Abstract

Linked Open Data initiatives have made available a diversity of scientific collections where scientists have annotated entities in the datasets with controlled vocabulary terms from ontologies. Annotations encode scientific knowledge, which is captured in annotation datasets. Determining relatedness between annotated entities becomes a building block for pattern mining, e.g. identifying drug–drug relationships may depend on the similarity of the targets that interact with each drug. A diversity of similarity measures has been proposed in the literature to compute relatedness between a pair of entities. Each measure exploits some knowledge including the name, function, relationships with other entities, taxonomic neighborhood and semantic knowledge. We propose a novel general-purpose annotation similarity measure called ‘AnnSim’ that measures the relatedness between two entities based on the similarity of their annotations. We model AnnSim as a 1–1 maximum weight bipartite match and exploit properties of existing solvers to provide an efficient solution. We empirically study the performance of AnnSim on real-world datasets of drugs and disease associations from clinical trials and relationships between drugs and (genomic) targets. Using baselines that include a variety of measures, we identify where AnnSim can provide a deeper understanding of the semantics underlying the relatedness of a pair of entities or where it could lead to predicting new links or identifying potential novel patterns. Although AnnSim does not exploit knowledge or properties of a particular domain, its performance compares well with a variety of state-of-the-art domain-specific measures.

Database URL: http://www.yeastgenome.org/

## Introduction

One of the early successes of the Linked Data initiatives is the publication of a diversity of scientific collections, e.g. Bio2RDF is the largest project of Linked Data for Life Sciences (https://github.com/bio2rdf/bio2rdf-scripts/wiki). Scientists have annotated entities in these collections with controlled vocabulary (CV) terms from ontologies or taxonomies. Annotations describe properties of these entities, e.g. the functions of genes are described using Gene Ontology (GO) CV terms and with the Resource Description Framework predicate drugbank: goClassificationFunction in the DrugBank dataset (http://wifo5-03.informatik.uni-mannheim.de/drugbank).

**Figure 1.**
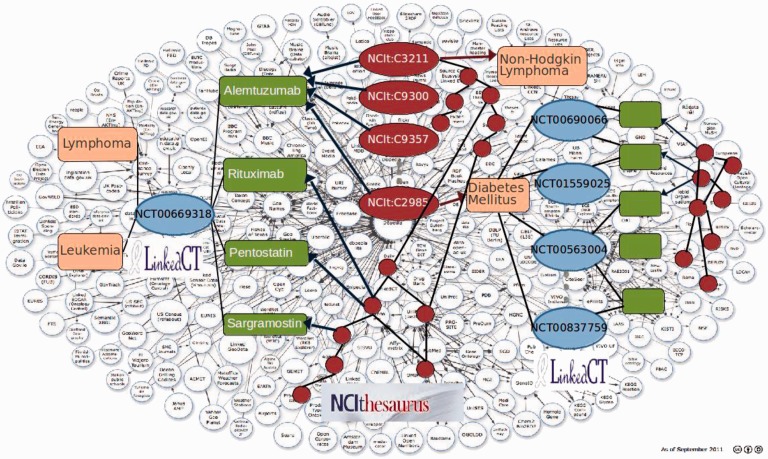
Annotation graph of Clinical Trials from LinkedCT (blue ovals). Interventions are green rectangles; conditions are pink rectangles and CV terms from the NCIt are red ovals.

Annotations induce an annotation graph where nodes correspond to scientific entities or ontology terms, and edges represent relationships between entities. Figure 1 illustrates a portion of the Linking Open Data cloud that induces an annotation graph. Consider clinical trials linked to a set of diseases or conditions in the NCI Thesaurus (NCIt). Clinical trials from LinkedCT (http://linkedct.org/) are represented by blue ovals; they are associated with interventions or drugs (green rectangles) and diseases or conditions (pink rectangles). Both interventions and conditions are then annotated with terms from the NCIt (red circles). Some annotations of a drug may correspond to terms in the NCIt that identify the drug, whereas others may correspond to the diseases or conditions that have been treated with this drug. Knowledge captured within scientific collections, annotations and ontologies are rich and complex. For example, the NCIt version 12.05d has 93 788 terms. The LinkedCT dataset *circa* September 2011 includes 142 207 interventions, 167 012 conditions or diseases and 166 890 links to DBpedia, DrugBank and Diseasome. Thus, the challenge is to explore these rich and complex datasets to discover patterns that will allow for the discovery of potential novel associations. For instance, Palma *et al*. (1) have proposed a novel edge partition technique that relies on semantic similarities to identify patterns across drug and target interactions; these patterns are further used to suggest novel interactions, which could be validated in latest online version of STITCH (http://stitch.embl.de/).

As a first step to discover complex patterns, we propose a similarity measure ‘AnnSim’ that determines the relatedness (or similarity) of a pair of scientific entities, based on their annotations with respect to one or more ontologies. An example is identifying the relatedness or similarity of (drug, drug) pairs, based on the annotation evidence of diseases (conditions) from the NCIt. Identifying relatedness between drugs can lead to discoveries of new targets for these drugs, or it can predict their potential side-effects.

A broad variety of similarity measures have been proposed in the literature, and they can be of diverse types. String-similarity measures compute similarity using string matching functions (e.g. Ref. 2), whereas path-similarity measures, such as ‘PathSim’ (3) and ‘HeteSim’ (4), compute relatedness based on the paths that connect entities in a graph. Structural or context-based measures determine if two entities are similar in terms of their relationships with other entities [e.g. SimRank (5)], whereas topological-similarity measures compute relatedness based on the closeness of CV terms in a given taxonomy or ontology (e.g. Refs. 6–8). Function or domain-specific measures reflect relatedness of entities based on their properties or function, e.g. Sequence Similarity relies on the Smith–Waterman scores (9). Ontological similarity measures exploit knowledge encoded in ontologies to compute the semantic similarity between terms (10–13), whereas Information Content (IC) measures rely on IC to compute similarity between entities (14–19).

We propose a measure named AnnSim that determines the relatedness of two entities in terms of the similarity or relatedness of (two sets of) their annotations. AnnSim combines properties of path- and topological-based similarity measures to decide the relatedness between these annotations. To the best of our knowledge, our research is the first to consider both the shared annotations between a pairs of entities of any abstract type, as well as the relatedness of the annotations (CV terms) within some ontology, to determine the resulting relatedness of the two entities.

**Example 1.1**

**Figure 2.**
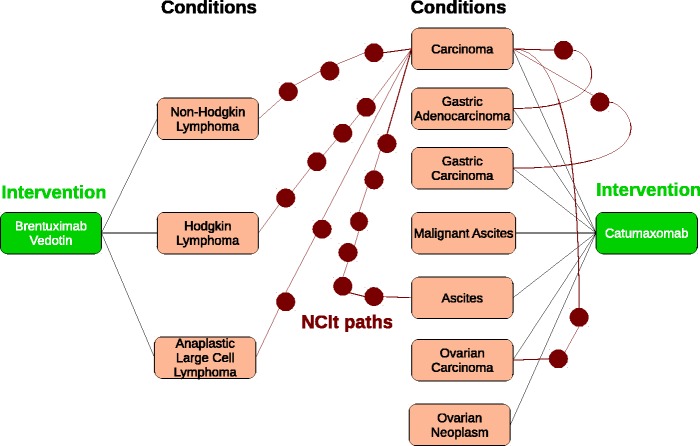
Annotation subgraph representing the annotations of Brentuximab vedotin and Catumaxomab. Interventions are green rectangles; conditions are pink rectangles and ontology terms in the NCIt are red circles. (**a**) Weighted bipartite graph for Brentuximab vedotin and Catumaxomab. (**b**) 1–1 maximum weight bipartite matching for Brentuximab vedotin and Catumaxomab

*Antineoplastic agents and monoclonal antibodies are two popular and independent intervention regimes that have been successfully applied to treat a large range of cancers. There are 12 drugs that fall within their intersection, and scientists are interested in studying the relationships between these drugs and the corresponding diseases. Consider the two drugs* Brentuximab vedotin *and* Catumaxomab. *Figure* *2* *represents an annotation graph of* *Figure* *1**. Each path between a pair of conditions, e.g.* Carcinoma *and* Anaplastic Large Cell Lymphoma *through the NCIt is identified using red circles*,* which represent ontology terms from the NCIt. The count of red circles represents the length of a path in NCIt. To simplify the figure, we only illustrate the paths from the term* Carcinoma.

We model AnnSim as a 1–1 maximum weight bipartite matching, and we exploit properties of existing solvers to provide an efficient solution. We empirically study the effectiveness of AnnSim on real-world datasets of evidences from clinical trials and a well known human disease benchmark. We compare the quality of AnnSim with respect to existing similarity measures including *d*_tax_ (7), *d*_ps_ (8), HeteSim (4) and semantic similarity measures (14–19).

Additionally, we use the online tool Collaborative Evaluation of Semantic Similarity Measures (CESSM) to compare AnnSim with respect to state-of-the-art semantic similarity measures. Finally, we evaluate AnnSim on two datasets comprising drugs, targets and interactions. The first dataset was collected by Perlman *et al*. (20) and comprises 310 drugs, 210 targets and 1306 interactions from DrugBank. The second dataset of drug–target interactions collected by Yamanishi *et al*. (21), and it comprises four subsets of Nuclear receptors, Gprotein-coupled receptors (GPCRs), Ion channels and Enzymes, obtained from KEGG BRITE (22), BRENDA (23), SuperTarget (24) and DrugBank (25). In both datasets, drugs and targets are associated with domain-specific similarity measures, and the goal of these experiments is to evaluate the behavior of a general-purpose measure as AnnSim with respect to state-of-the-art domain-specific measures, as well as the potential of uncover patterns that may lead to the discovery of new relationships and interactions.

This article extends the work by Palma *et al*. (26). Our contributions are summarized as follows:

- The formalization of an annotation-based similarity measure AnnSim that defines the relatedness of two entities in terms of the sets of their annotations. AnnSim is a general-purpose measure that exhibits a stable behavior in a variety of scientific entities and ontologies. The implementation of AnnSim is built on top of an existing 1–1 maximum weight bipartite matching solver.
- An empirical study that validates properties and behavior of AnnSim using a variety of ground truth datasets including human curation. Empirical analysis of the experimental results suggests that AnnSim can provide a deeper understanding of the relatedness of entities, and in some cases, it can also provide an explanation of patterns.
- The evaluation of the correlation of AnnSim with respect to the sequence similarity measure (9) and the comparison of this behavior with respect to state-of-the-art semantic similarity measures (14–19). Reported results were produced by the online tool CESSM and reveal that AnnSim is competitive even with combined and domain-specific measures that consider both IC and structural characteristics of the compared annotations.
- An empirical study to compare the behavior of AnnSim with respect to several state-of-the-art domain-specific measures for drugs and targets. The evaluation consists on the generation of clusterings of the drugs based on drug–drug similarity measures and AnnSim. The data mining WEKA tool is used to generate the clusterings, and diverse measures are computed to measure the quality of the clusterings. The study shows that the clusterings of drugs based on AnnSim can be used to uncover patterns that suggest potential new associations between drugs and targets.

This article is organized as follows: Section ‘Related work’ summarizes related work and gives the preliminary knowledge of this work and illustrates the performance of existing approaches in a real-world example. Section ‘Annotation similarity measure for annotation graphs’ presents our approach. Experimental results are reported in Section ‘Experimental evaluation’. Finally, we conclude in Section ‘Conclusions and future work’ with an outlook to future work.

## Related work

Determining relatedness between entities becomes a building block for pattern mining. A diversity of similarity measures has been proposed in the literature to compute relatedness between a pair of entities. Each measure exploits some knowledge including the name, function, relationships with other entities, taxonomic neighborhood and semantic knowledge. We classify existing measures as string-, path-, graph-based, functional or domain-specific or semantics-based similarity measures. We also describe different techniques that rely on graph matching algorithms to compute the values of similarity.

### String-based similarity measures

The first class of measures include string similarity; they compare names or labels of entities using string comparison functions based on edit distances or other functions that compare strings. The broadly used string distance measures either reflect the number of edit operations that have to be performed on two strings to convert one into the other (e.g. the Levenstein distance) or they count the number and order of common characters between two strings [e.g. Jaro-Winkler (2)].

### Path- and structure-based similarity measures

Path- or structure-based similarity measures compute the relatedness of two entities according to the properties of the paths that connect them [e.g. PathSim (3) or HeteSim (4) or *d*_ps_ (8) or *d*_tax_ (7)] or the structure of the graph that includes the two entities [e.g. SimRank (5), nan (6)]. Entities in the paths can be all of the same abstract types [e.g. PathSim (3)] or they can be heterogeneous [e.g. HeteSim (4)]. Further, similarity between entities in a graph can be measured recursively in terms of the similarity of their neighbors, e.g. SimRank (5). High values of structure-based similarity indicate that the entities are connected with a large number of paths that meet certain conditions or the sub-graph that includes both entities is highly connected.

We consider details of a few measures. *d*_tax_ (7) and *d*_ps_ (8) define the distance of two nodes in terms of the depth of the nodes to the root of the ontology and the distance to the their lowest common ancestor (LCA). These concepts are defined as follows: given a directed acyclic graph G, the depth of a vertex *x* in G is the length of the longest path from a root of G to *x*. Given a directed acyclic graph G, the ‘lowest common ancestor’ (27) of two vertices *x* and *y* is the vertex of greatest depth in G that is an ancestor of both *x* and *y*. Let *d*(*x*, *y*) be the number of edges in the shortest path between vertices *x* and *y* in a given ontology. Also let lca(*x*, *y*) be the LCA of vertices *x* and *y*.

The intuition behind the *d*_ps_ measure is to capture the ability to represent the taxonomic distance between two vertices with respect to the depth of the common ancestor of these two vertices. Extending on this idea, *d*_tax_ (7) assigns low(er) values of taxonomic distance to pairs of vertices that are (i) at greater depth in the taxonomy and (ii) they are closer to their LCA. A value close to 0.0 means that the two vertices are close to the leaves and both are close to their LCA. A value close to 1.0 represents that both vertices are general or that the LCA is close to the root of the taxonomy. The distance measure *d*_tax_ is as follows where, root is the root node in the ontology:

(1) $$d_{\text{tax}}(x,y)=\frac{d(\text{lca}(x,y),x)+d(\text{lca}(x,y),y)}{d(\text{root},x)+d(\text{root},y)}$$

The distance measure *d*_ps_ is defined as follows:

(2) $$d_{\text{ps}}(x,y)=1-\frac{d(\text{root,lca}(x,y))}{d(\text{root,lca}(x,y))+d(\text{lca}(x,y),x)+d(\text{lca}(x,y),y)}$$

The pair of drugs Brentuximab vedotin and Catumaxomab appears in the NCIt with codes C66944 and C62445, respectively. Thus, we could use either of the distance measures and compute similarity values, we can use either of the path-based distance measures (1 − *d*_tax_) or (1 − *d*_ps_); the similarity values are 0.60 and 0.43, respectively. Note that unlike the proposed AnnSim measure, this similarity between the pair of drugs only considers their location within the NCIt and does not exploit knowledge of their annotations, e.g. the diseases associated with these drugs.

The measure HeteSim (4) defines the relatedness of entity pairs in terms of the paths that connect the entities in a graph. Paths considered during the computation of this measure are type-path constrained, i.e. they must correspond to instances of a sequence of classes or types named relevance path. HeteSim(*s,t*|*P*) measures how likely *s* and *t* will meet at the same entity when *s* follows along the path that respects the relevance path *P* and *t* goes against the path. Shi *et al*. (4) define a relevance path as a meta-path that encodes the conditions to be met by the paths that are considered in the computation of the measure, i.e. a composite relation where HeteSim is computed.

**Definition 2.1**

[*Relevance Path* (4)] *Given a schema S = *(*A, R*)*, where A and R are sets of entity and relation types, respectively. A relevance path of the form* $P=A_{1}\overset{R_{1}}{\to }A_{2}\overset{R_{2}}{\to }\cdots \overset{R_{l}}{\to }A_{l+1}$ *corresponds to a composite relation R = R*_1_ ○ *R*_2_ ○ ⋯ ○ *R*_l_ *between entity types A*_1_ *and A*_l+1_*, where* ○ *denotes the composition operator between relation types. The number of relation types in the path indicates the length of the path.*

**Definition 2.2**

[*HeteSim* (4)] *Given two objects s and t* (*s* ∈ *R*_1_ *and t* ∈ *R*_l_) *and a relevance path* $P=A_{1}\overset{R_{1}}{\to }A_{2}\overset{R_{2}}{\to }\cdots \overset{R_{l}}{\to }A_{l+1}$ *that corresponds to a composite relation R = R*_1_ ○ *R*_2_ ○ ⋯ ○ *R*_l_*,*

HeteSim(s,t|R1○R2○⋯○Rl)=1|O(s|R1)||I(t|Rl)∑i=1|O(s|R1)|∑j=1|I(s|Rl)|HeteSim(Oi(s|R1),Ij(t|Rl)|R2○⋯○Rl−1)

where $O(s|R_{i})$ and $I(s|R_{j})$ correspond to the out-neighbors and in-neighbors of *s* based on relations *R_i_* and *R_j_*, respectively, and $O_{t}(s|R_{i})$ and $I_{k}(s|R_{j})$ represent the *t*th and *k*th elements in the out-neighbors and in-neighbors of s based on relations *R_i_* and *R_j_*, respectively.

For example, given the annotation graph of Figure 2 and paths of type (Drug, NCIt, NCIt, Drug), HeteSim(Brentuximab vedotin, Catumaxomab) has a value of 0.0; this is because HeteSim only considers an exact match between the NCIt annotations of each drug. We note that HeteSim could be extended to further consider paths through the NCIt, i.e. these will be paths outside the annotation dataset.

### Conceptual similarity measures

In addition to the name of an entity or its position in an ontology or neighborhood, the semantics encoded in an ontology can also be considered to compute relatedness. Conceptual similarity measures assign a value of similarity to two entities based on a given ontology. They extend path similarity and consider relationships captured within an ontology or taxonomy [e.g. nan (6), *d*_ps_ (8) and *d*_tax_ (7)]. The intuition is that ontology terms that are located in proximity and are farther from the root are more related. Further, entities which share a LCA that is close to them are also considered similar.

### Functional and domain similarity measures

In the context of Biomedicine, domain-specific similarity measures have been defined to measure relatedness between entities of a specific abstract type, e.g. between drugs or genes. Smith and Waterman (28) propose an algorithm to identify sequence alignment in sequences of nucleotides or amino acids. BLAST (http://blast.ncbi.nlm.nih.gov/) and FASTA (http://www.ebi.ac.uk/Tools/sss/fasta/) propose some restrictions to the sequence entries to speed up the alignment computation process, potentially at the cost of reducing quality. Furthermore, domain-specific annotation-based measures rely on knowledge encoded in specific taxonomies or ontologies to compute the similarity of two entities. The GO semantic similarity measures assign values between GO annotation terms of targets according to the similarity measures proposed by Resnik (29), Lin (15) and Jiang and Conrath (14). Similarly, the World Health Organization (WHO) annotation-based similarity considers the WHO Anatomical, Therapeutic and Chemical (ATC) classification system (20) to compute values of similarity between drugs. Furthermore, Othman *et al*. (30) use shared annotations of GO with the aim of obtaining a set of GO terms that have higher term similarity scores for these GO terms. Nevertheless, the proposed approach is not able to determine similarity of two sets of GO terms, and in consequence, it can miss structural relatedness across the set of annotations.

Hao Ding *et al*. (31) evaluate the impact of domain-specific drug–drug and target–target similarity measures and state-of-the-art machine learning techniques in the accuracy of predicting interactions between drugs and targets. The studied approaches rely on the assumption that similar drugs interact with similar targets, and the reported results suggest that using domain-specific measures allow to identify drugs and targets that meet this assumption and thus, identify potential new interactions. Similarly, Zheng *et al.* (32) present a machine learning-based technique that relies on existing biomedical similarity measures to predict interactions between drugs and targets. To conclude the results reported by Perlman *et al.* (20), Hao Ding *et al*. (31) and Zheng *et al*. (32) suggest that that existing biomedical similarity measures can precisely measure relatedness; nevertheless, small changes in the ontologies or controlled vocabularies used to annotate the entities may affect their behavior. In contrast, we propose a general measure that exploits knowledge encoded in the annotations and exhibits a stable behavior for scientific entities of a variety of abstract types and properties.

Recently, Couto and Pinto (33) study biomedical ontologies and propose a classification of similarity measures according to the type of meaning they are able to consider. Terminological measures compute relatedness between two entities by considering similarity between the names of the classes to which these entities belong, whereas structural approaches decide similarity depending on the relationships and attributes of the classes. Furthermore, extensional measures compute similarity based on the cardinality of the intersection of the instantiations of the classes, and the semantic-based approaches take into account axioms that formalize properties of ontology classes to decide relatedness of two entities. Additionally, Couto and Pinto (33) reinforce the statement stated by d’Amato *et al*. (34) that establishes limitations of the structural and extensional measures in considering semantics encoded in axioms of equivalence and disjunction. We propose a conceptual similarity measure that decides similarity of two entities based on the perfect matching of the annotations of the entities. Structural measures are used to decide if two annotations match or not. Thus, based on Couto and Pinto (33) classification, AnnSim is a structural measure. Nevertheless, if a semantic-based measure were used to compare the annotations, AnnSim is able to overcome limitations of structural approach and can be considered a semantic-based similarity measure.

### Graph match to compute similarity measures

There have been several solutions using graph match to compute the similarity of two entities based on their neighborhood graph. Thiagarajan *et al*. (35) compute relatedness in terms of a bag of terms that describes each of these entities. Relationships between the terms are represented as a bipartite graph where edges are annotated with the length of the path between each of the terms in the two bags. Similarity is computed as the optimal bipartite matching of the bipartite graph based on the length of the paths. Furthermore, the problem of 1–1 maximum weight bipartite matching has been tested on specific domains, e.g. semantic equivalence between two sentences and measuring similarity between shapes for object recognition (36–38). Belongie *et al*. (36) measure the similarity between two shapes; this is computed as the transformation that best aligns the shapes. Bhagwani *et al*. (37) find the similarity of two sentences assuming that a sentence includes one or multiple words. The similarity between words is measured using the Lin similarity measure (15) and the is-a hierarchy of WordNet. Shavitt *et al*. (38) propose a measure for peer similarity on peer-to-peer (p2p) networks.

Although these approaches rely on the computation of the 1–1 maximum weight bipartite match, they do not consider information about the structural similarity of each of the pairs of terms that comprise the bipartite graph. AnnSim differs from them since it does consider the relatedness of the sets of annotations or terms. It uses an ontology structure to determine ontological relatedness and extends the dice coefficient to measure set agreement between the sets of annotations in the 1–1 maximum weight bipartite matching. The AnnSim score will be penalized if one of the entities is associated with a large number of annotations, while only a small number of annotations participate in the match. Finally, we note that the value of any annotation-based similarity measure will naturally depend on the accuracy and comprehensiveness of the underlying annotation, i.e. if the annotations are not negligibility, inaccurate or subjective (33). As AnnSim considers the graph structure of the ontology, it has the potential to be robust and stable in the presence of missing or incomplete annotations, or similar yet not identical annotations.

## Annotation similarity measure for annotation graphs

In this section, we present AnnSim, a similarity measure for entities of an annotation graph. An annotation graph *G = *(*V, E*) is a particular graph comprising two type of nodes in *V*: scientific entities and terms from an ontology. Edges in *G* can be between scientific entities and ontology terms.

Given two entities *c*_1_ and *c*_2_ from an annotation graph *G = *(*V*, *E*), we define an annotation similarity measure, AnnSim, based on their sets of annotations, *A*_1_ and *A*_2_, respectively. We assume that we know the pairwise similarity between elements of *A*_1_ and elements of *A*_2_, i.e. sim(*a*_1_, *a*_2_) ∈ [0, 1] for all *a*_1_ ∈ *A*_1_ and *a*_2_ ∈ *A*_2_. These relationships between terms in *A*_1_ and *A*_2_ can be represented as a weighted bipartite graph *BG* with two node sets *A*_1_ and *A*_2_. An edge between *a*_1_ ∈ *A*_1_ and *a*_2_ ∈ *A*_2_ has a weight sim(*a*_1_, *a*_2_), where sim(*a*_1_, *a*_2_) is computed using a taxonomic distance measure.

The computation of AnnSim first requires building a bipartite graph *BG* with the links in the Cartesian product between the set of annotations of two scientific entities, computing all pairwise similarities and then determining the 1–1 maximum weight bipartite matching. The time complexity of computing the 1–1 maximum weight bipartite matching is *O*(*m*^4^), where *m* is sum of the cardinalities of *A*_1_ and *A*_2_. Although the cost of computing the topological similarity values of each pair of terms is *O*(*n*^2^), where *n* is the number of nodes in the ontology. To achieve an efficient implementation of AnnSim, we reduce the bipartite graph *BG* to a ‘1–1 maximum weight bipartite matching MWBG’.

**Definition 3.1**

(39) A *1–1 maximum weight bipartite matching MWBG = *(*A*_1_ *∪* *A*_2_*, WEr*) *for a weighted bipartite graph BG = *(*A*_1_ *∪* *A*_2_*, WE*) *is as follows:*

- *WEr* ∈ *WE, i.e.* *MWBG is a sub-graph of BG.*
- *the sum of the weights of the edges in WEr is maximized, i.e*.
  $$\text{max}\sum_{(a_{1},a_{2})\in WE}\text{s}\text{im}(a_{1},a_{2})$$
   
- *for each node in A*_1_ *∪* *A*_2_ *there is only one incident edge in WEr, i.e*.
  – $\sum_{i=1}^{|A_{1}|}\left(a_{i},a_{j}\right)=1,\forall j=1\ldots |A_{2}|$
  – $\sum_{j=1}^{|A_{2}|}\left(a_{i},a_{j}\right)=1,\forall i=1\ldots |A_{1}|$

**Example 3.1**

**Figure 3.**
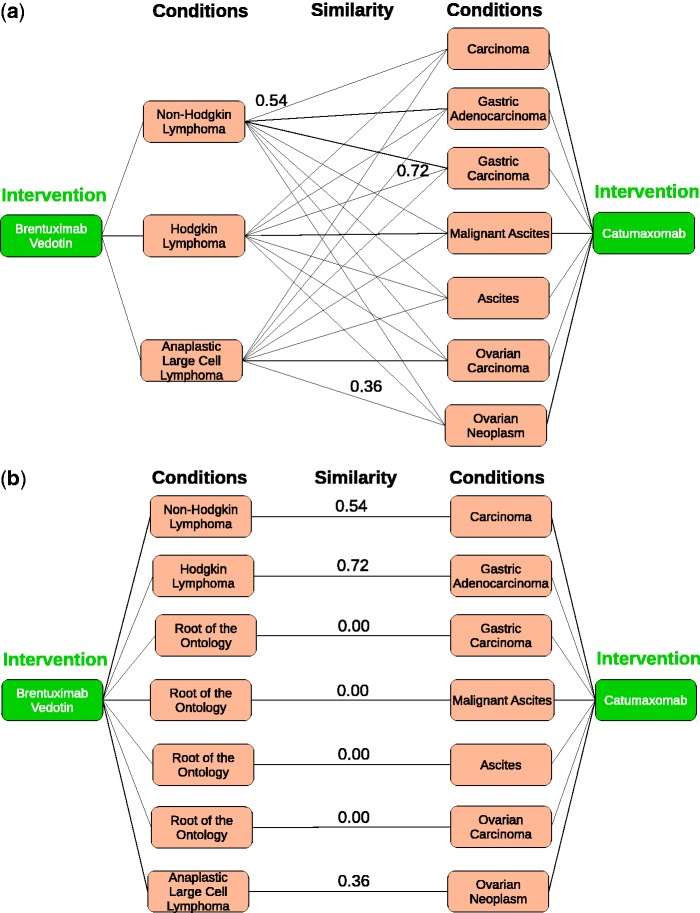
Bipartite graphs for drugs Brentuximab vedotin and Catumaxomab. For legibility, only the value of the highest matching edges is shown in (**a**). (a) Weighted bipartite graph for Brentuximab vedotin and Catumaxomab. (**b**) 1-1 maximum weight bipartite matching for Brentuximab vedotin and Catumaxomab.

*Consider the two drugs* Brentuximab vedotin *and* Catumaxomab. *Figure* *3* *represents the 1–1 maximum weight bipartite matching produced by the BlossomIV solver* (*40*).

**Definition 3.2**

(*AnnSim Annotation Similarity*) *Consider two entities c*_1_ *and c*_2_ *annotated with the set of terms A*_1_ *and A*_2_ *in an annotation graph G. Let BG = *(*A*_1_ *∪* *A*_2_*, WE*) *be a weighted bipartite graph for set of terms A*_1_ *and A*_2_*. Let MWBG = *(*A*_1_ *∪* *A*_2_*, WEr*) *be 1–1 maximum weight bipartite matching for BG. The annotation similarity of c*_1_ *and c*_2_ *is defined as follows:*

$$\text{AnnSim}(c_{1},c_{2})=\frac{2\cdot \sum_{(a_{1},a_{2})\in WEr}\text{sim}(a_{1},a_{2})}{\left|A_{1}|+|A_{2}\right|}$$

The above definition is in the style of the well-known Dice coefficient. The maximal similarity of 1.0 is achieved if and only if both annotation sets have the same cardinality ($\left|A_{1}|=|A_{2}\right|$) and all edge weights equal 1. Further, AnnSim penalizes (large) differences in the cardinality of *A*_1_ and *A*_2_. We apply an exact solution to the problem of computing the 1–1 maximum weight bipartite matching MWBG from a weighted bipartite graph *BG* using the BlossomIV solver (40). To illustrate our proposed solution, consider the bipartite graph in Figure 3a where conditions correspond to the annotations of the drugs Brentuximab vedotin and Catumaxomab. Edges in the bipartite graph are labeled with values of a given taxonomic similarity measure that computes similarity of the NCIt terms associated with these conditions. For example, a value of 0.714 between Hodgkin Lymphoma and Gastric Carcinoma indicates that the NCIt terms corresponding to these two conditions are specific terms and share a LCA, which is also relatively far from the most general terms of the NCIt, i.e. the path between the LCA of the NCIt terms for the conditions Hodgkin Lymphoma and Gastric Carcinoma has a length greater than one. Values of similarity between conditions are used to compute the 1–1 maximum weight bipartite matching. Figure 3b presents the 1–1 maximum weight bipartite matching for anticancer drugs Brentuximab vedotin and Catumaxomab produced by the BlossomIV solver (40). We can observe that in the best matching, the sum of the similarity of the edges is maximized. Once the 1–1 maximum weight bipartite matching is produced, AnnSim is computed as indicated in Definition 3.2. For the 1–1 maximum weight bipartite matching of the drugs Brentuximab vedotin and Catumaxomab, AnnSim is 0.324 representing certain grade of similarity between these two drugs.

**Theorem 3.1**

(*Properties of AnnSim*) *Consider two entities c*_1_ *and c*_2_ *annotated with the set A*_1_ *and A*_2_ *in an annotation graph G then:*

- *Symmetry: AnnSim*(*c*_1_*,* *c*_2_) *=* *AnnSim*(*c*_2_*,* *c*_1_)*.*
- *Self-maximum: AnnSim*(*c*_1_, *c*_2_) ∈ [0, 1].
- Time complexity: polynomial in the size of G.

## Experimental evaluation

We provide details of the datasets and our protocol to construct ground truth datasets for evaluation. We then present evaluation results. The goal of the evaluation is to analyze the benefits of the knowledge encoded in the annotations that is exploited by AnnSim for a variety of domains. Table 1 summarizes the datasets. AnnSim source code, the datasets from Table 1, and instructions for to conduct the experiments in this section, can be obtained at https://code.google.com/p/annsim/. Table 2 summarizes the characteristics of the ontologies used in the evaluation datasets.

**Table 1.** Description of the datasets

Dataset	Description
1	Thirty pairs of diseases from the Mayo Clinic benchmark
2	Twelve anticancer drugs in the intersection of monoclonal antibodies and antineoplastic agents
3	Collection of pairs of proteins from UniProt^a^
4	Collection of drugs and targets interactions from DrugBank,^b^ introduced by Perlman *et al*. (20)
5	Collection of drug and target interactions collected by Yamanishi *et al*. (21)

^a^http://www.uniprot.org/.

^b^http://www.drugbank.ca/.

**Table 2.** Description of the ontologies used in the evaluation datasets

Ontology	NCIt	SNOMED CT	MeSH	GO
Version	12.05d	June 2012	June 2012	August 2008
Number of nodes	93 788	395 346	26 580	26 539
Number of arcs	104 439	539 245	36 212	43 213
Used in Dataset	1 and 2	1	1	3

### Datasets and evaluation roadmap

**Dataset 1**

Thirty pairs of diseases from the Mayo Clinic Benchmark; each pair is coded for similarity from 1.0 (least similar) to 4.0 (most similar). The coding was performed by 3 physicians (Phy) and 10 medical coders from the Mayo Clinic (Cod) (6, 41). Diseases were annotated with NCIt version 12.05d. Dataset 1 is used to compare (1 − *d*_tax_) and (1 − *d*_ps_) using SNOMED and MeSH.

**Dataset 2**

Twelve anticancer drugs in the intersection of monoclonal antibodies and antineoplastic agents: Alemtuzumab, Bevacizumab, Brentuximab vedotin, Cetuximab, Catumaxomab, Edrecolomab, Gemtuzumab, Ipilimumab, Ofatumumab, Panitumumab, Rituximab and Trastuzumab. The drugs were associated with conditions or diseases in clinical trials in LinkedCT *circa* September 2011 and each disease was linked to its corresponding term in the NCIt version 12.05d. The number of annotations varies from 1 to 100+. Dataset 2 is used to compare AnnSim with (1 − *d*_tax_), (1 − *d*_ps_) and HeteSim. We recognize that HeteSim performs poorly because it is not designed to consider terms that are close to each other in the ontology as related. However, we use this baseline since it is the only measure that can consider paths between nodes of different types, i.e. drugs and diseases.

**Dataset 3**

This corresponds to the CESSM collection that is published through the site http://xldb.di.fc.ul.pt/tools/cessm/. This collection contains pairs of proteins from UniProt (http://www.uniprot.org/); they are annotated with GO terms separated into the GO hierarchies of biological process (BP), molecular function and cellular component. GO and UniProt are both from August 2008. The similarity of the pairs of proteins as measured by 11 similarity measures described in Table 3 are available. Dataset 3 is used to obtain the Pearson’s correlation for AnnSim with respect to ‘EC’ similarity (44), ‘Pfam’ similarity (45) and sequence similarity ‘SeqSim*’* (9). The correlation coefficient of AnnSim will be compared with the correlation coefficients of 11 semantic similarity measures for the three standards of evaluation: EC, Pfam and SeqSim.

**Table 3.** Similarity measures for pairs of proteins in dataset 3

simUI (UI)	Jaccard index on the GO annotations of the proteins.
simGIC (GI) (17)	Jaccard index where GO annotations of the compared proteins are weighted by their IC.
Resnik (18, 29) Average (RA)	Resnik’s measure where similarity of two terms is the average of IC of pairs of common ancestors.
Resnik (29, 42) Maximum (RM)	Resnik’s measure where similarity corresponds to the maximum value of IC of pairs of common ancestors.
Resnik (29, 43) Best-Match Average (RB)	Resnik’s measure where similarity corresponds to the average of IC of pairs of disjunctive common ancestors (DCA).
Lin (15, 18) Average (LA)	Lin’s measure that relates IC of the average of IC of pairs of common ancestors to IC of compared terms.
Lin (15, 42) Maximum (LM)	Lin’s measure that relates IC of the maximum value of IC of pairs of common ancestors to IC of compared terms.
Lin Best-Match (15, 43) Average (LB)	Lin’s measure that relates the IC of the average of the IC of pairs of DCA to IC of compared terms.
Jiang and Conrath (18, 14) Average (JA)	Jiang and Conrath’s measure where IC of average of IC of pairs of common ancestors is related to IC of compared terms.
Jiang and Conrath (14, 42) Maximum (JM)	Jiang and Conrath’s measure where IC of the maximum IC of pairs of common ancestors is related to IC of compared terms.
Jiang and Conrath (14, 43) Best-Match Average (JB)	Jiang and Conrath’s measure where the IC of the average IC of pairs of DCA is related to IC of compared terms.

**Dataset 4**

This corresponds to a collection of interactions between drugs and targets from DrugBank (http://www.drugbank.ca/). This dataset was collected by Perlman *et al*. (20) and comprises 310 drugs, 210 targets and 1306 interactions as table 4 shows. Both drugs and targets are associated with domain-specific similarity measures; there are five measures for drug–drug pairs and three measures for target–target pairs, as described in Table 5. Dataset 4 is used to evaluate the quality of AnnSim with respect to a gold standard drug–drug similarity measure.

**Table 4.** Statistics of dataset 4 obtained from Perlman *et al*. (20)

Number of drugs	Number of targets	Number of drug–target interactions
315	250	1306

**Table 5.** Similarity measures for drugs and targets in dataset 4 (20)

Drug–drug similarity measures	
Chemical based	Jaccard similarity of the SMILES fingerprints of the drugs.
Ligand based	Jaccard similarity between protein receptor families extracted via matched ligands with drugs’ SMILES on the SEA tool.
Expression based	Spearman’s correlation of gene expression responses to drugs using connectivity map.
Side-effect-based	Jaccard similarity between drugs side-effects from SIDER.
Annotation based	Semantic similarity of drugs based on the WHO ATC classification system.
Target–target similarity measures	
Sequence based	Smith and Waterman scores (9) computed by BLAST^a^ and normalized as suggested in Ref. 46.
Protein based	Shortest paths between human protein–protein interactions of the drugs.
GO based	Semantic similarity based on GO annotations computed using csbl.go package of R.^b^

^a^http://blast.ncbi.nlm.nih.gov/.

^b^http://csbi.ltdk.helsinki.fi/csbl.go/.

For each pair of drugs in Dataset 4, we compute AnnSim with respect to the set of associated targets, i.e. the targets are interpreted as the annotations of the drugs. The target–target similarity measures are also considered by AnnSim.

The gold standard for the similarity of two drugs is based on the Jaccard Index (47) of the categories of the drugs published by DrugBank, i.e. the size of the intersection divided by the size of the union of the set of categories. In DrugBank, drug categories correspond to therapeutic or general categories manually collected from PubMed (http://www.ncbi.nlm.nih.gov/pubmed/), STAT!Ref (AHFS) (http://online.statref.com/UserLogin.aspx?Path=/Default. aspx&Product=StatRef) and e-Therapeutics (http://www.e-therapeutics.ca/).

**Dataset 5**

Collection of drug and target interactions used in the experimental study reported by Hao Ding *et al*. (31). The dataset comprises four subsets of nuclear receptors, GPCRs, ion channels and enzymes; this data are obtained from KEGG BRITE (22), BRENDA (23), SuperTarget (24) and DrugBank (25). Pairs of drugs are associated with similarity computed from the chemical structures of drugs [obtained from KEGG LIGAND (22)] by using SIMCOMP (48). Target similarity corresponds to target sequences [obtained from KEGG GENES (23)] by using a normalized Smith–Waterman score (9). As with Dataset 4, this dataset is used to evaluate the quality of AnnSim with respect to a well-known drug–target gold standard. Table 6 shows statistics of the dataset 5.

**Table 6.** Statistics of dataset 5 downloaded from http://web. kuicr.kyoto-u.ac.jp/supp/yoshi/drugtarget/ (21)

Statistics	Nuclear receptor	GPCR	Ion channel	Enzyme
Number of drugs (D)	54	23	210	445
Number of targets (T)	26	95	204	664
Number of D-T interactions	90	635	1476	2926

### Effectiveness in dataset 1

The goal of the experiment is to tune the performance of (1 − *d*_tax_) and (1 − *d*_ps_) with respect to multiple ontologies. This study will reveal if AnnSim scores will be stable across different taxonomic measures and ontologies.

We annotated the 30 diseases of Dataset 1 with their corresponding terms in SNOMED, MeSH and the NCIt. Table 7 shows all pairs of diseases. The scores determined by (1 − *d*_tax_) and (1 − *d*_ps_) are compared with the human ground truth evaluation of physicians and coders. Table 8 reports on this comparison. Additionally, Table 9 reports on the Normalized Discounted Cumulative Gain (49) (nDCG) between the ranking of the results using (1 − *d*_tax_) and (1 − *d*_ps_) and the ground truth from a physician panel or a coder panel. The nDCG correlations take values between 0.0 and 1.0, where a value close to 1.0 represents a high correlation of the ranking induced by the similarity measure and the one in the ground truth.

**Table 7.** Identifiers of the 30 pairs of diseases from the Mayo Clinic benchmark

ID	Medical terms
1	Renal insufficiency – kidney failure
2	Heart – myocardium
3	Stroke – infarction
4	Abortion – miscarriage
5	Delusions – schizophrenia
6	Congestive heart failure – pulmonary edema
7	Metastasis – adenocarcinoma
8	Calcification – stenosis
9	Diarrhea – stomach cramps
10	Mitral stenosis – atrial fibrillation
11	Chronic obstructive pulmonary disease – lung infiltrates
12	Rheumatoid arthritis – lupus
13	Brain tumor – intracranial hemorrhage
14	Carpal tunnel syndrome – osteoarthritis
15	Diabetes mellitus – hypertension
16	Acne – syringe
17	Antibiotic – allergy
18	Cortisone – total knee replacement
19	Pulmonary embolism – myocardial Infarction
20	Pulmonary fibrosis – lung Cancer
21	Cholangiocarcinoma – colonoscopy
22	Lymphoid hyperplasia – laryngeal cancer
23	Multiple Sclerosis – psychosis
24	Appendicitis – osteoporosis
25	Rectal polyp – aorta
26	Xerostomia – liver cirrhosis, alcoholic
27	Peptic ulcer – myopia
28	Depression – cellulitis
29	Varicose vein – entire knee meniscus
30	Hyperlipidemia – metastasis

**Table 8.** Similarity dataset 1: (1 – *d*_tax_) and (1 – *d*_ps_) for SNOMED, MeSH and NCIt

ID	Phy	Cod	SNOMED		MeSH		NCIt	
			1 – *d*_tax_	1 – *d*_ps_	1 – *d*_tax_	1 – *d*_ps_	1 – *d*_tax_	1 – *d*_ps_
1	**4.00**	**4.00**	**1.00**	**1.00**	**1.00**	**1.00**	**1.00**	**1.00**
2	**3.30**	3.00	**0.77**	0.64	**0.80**	0.67	0.20	0.11
3	**3.00**	2.80	0.31	0.31	**0.80**	0.67	**0.87**	**0.78**
4	3.00	**3.30**	**0.89**	**0.80**	0.00	0.00	**0.92**	**0.86**
5	3.00	2.20	0.00	0.00	0.00	0.00	0.80	0.67
6	3.00	**1.40**	0.50	0.46	0.00	0.00	0.59	**0.42**
7	**2.70**	**1.80**	**0.83**	**0.71**	**0.25**	0.14	0.00	0.00
8	2.70	**2.00**	**0.55**	0.38	0.00	0.00	0.40	0.25
9	2.30	**1.30**	0.29	**0.17**	0.75	0.63	0.42	0.30
10	**2.30**	1.30	**0.63**	0.46	0.50	0.33	0.53	0.36
11	**2.30**	1.90	0.70	**0.63**	—	—	0.13	0.07
12	**2.00**	**1.00**	**0.50**	0.33	**0.00**	0.11	0.86	0.75
13	2.00	**1.30**	0.63	0.57	0.63	0.50	**0.17**	0.09
14	2.00	**1.00**	0.33	0.33	**0.00**	**0.00**	0.33	0.20
15	**2.00**	**1.00**	0.64	**0.50**	**0.00**	**0.00**	0.17	0.09
16	2.00	**1.00**	**0.00**	**0.00**	**0.00**	**0.00**	**0.00**	**0.00**
17	1.70	**1.00**	**0.00**	**0.00**	**0.00**	**0.00**	**0.00**	**0.00**
18	1.70	**1.00**	**0.00**	**0.00**	**0.00**	**0.00**	**0.00**	**0.00**
19	**1.70**	1.20	0.36	**0.42**	0.29	0.29	0.63	**0.46**
20	1.70	1.40	0.75	0.63	0.67	0.50	0.60	0.50
21	1.30	**1.00**	**0.00**	**0.00**	**0.00**	**0.00**	**0.00**	**0.00**
22	1.30	**1.00**	0.43	0.33	**0.00**	**0.00**	0.36	0.22
23	**1.00**	**1.00**	0.44	0.29	**0.00**	**0.00**	0.33	0.20
24	**1.00**	**1.00**	0.31	0.31	**0.00**	**0.00**	0.50	0.36
25	**1.00**	**1.00**	**0.00**	**0.00**	—	—	**0.00**	**0.00**
26	**1.00**	**1.00**	**0.00**	**0.00**	**0.00**	**0.00**	0.14	0.08
27	**1.00**	**1.00**	0.23	0.29	**0.00**	**0.00**	0.15	0.08
28	**1.00**	**1.00**	**0.00**	**0.00**	**0.00**	**0.00**	0.31	0.18
29	**1.00**	**1.00**	0.13	0.07	—	—	**0.00**	**0.00**
30	**1.00**	**1.00**	0.33	0.20	**0.00**	**0.00**	**0.00**	**0.00**

Empty cells (—) represent terms that do not appear in the ontology. Values highlighted in bold show high correlation between the relevance given by the physician, coder and the measures. IDs are presented in Table 7

**Table 9.** nDCG of (1 – *d*_tax_) and (1 – *d*_ps_)

Measure	SNOMED		MeSH		NCIt	
	Physician	Coder	Physician	Coder	Physician	Coder
1 − *d*_tax_	0.837	0.961	0.977	0.957	0.959	0.959
1 − *d*_ps_	0.966	0.963	0.976	0.987	0.959	0.959

Given the order of the pairs of diseases induced by the values of (1 − *d*_tax_) and (1 − *d*_ps_), a high value of nDCG of a given pair highly ranked by the physicians (or coders) indicates that the pair appears at the top of the ranking list. A low value of nDCG reflects that the relevant pair appears at the bottom of the ranking list. We can observe that both (1 − *d*_tax_) and (1 − *d*_ps_) have similar values of nDCG across SNOMED, MeSH and NCIt, for both physicians and coders. This reveals that both measures are successful at computing high values of similarity for the pairs that were also ranked highly by the physicians and coders. These values also suggest that both measures have similar performance.

To summarize, the two measures to compare taxonomic relatedness perform well across multiple ontologies, and their performance is matched.

### Effectiveness in dataset 2

The goal of this experiment is to study the impact of using the structural knowledge of shared annotations between two entities versus just considering the structural knowledge of these entities; we evaluate the impact of the NCIt annotations of drugs on Dataset 2 on the values of similarity. When all the drugs belong to the same family, a good similarity measure should assign high values of pair-wise similarity. We consider both topological measures (1 − *d*_tax_), (1 − *d*_ps_) and HeteSim to study the effects of the structural information of the entities. Intuitively, HeteSim would detect that two drugs are similar if they have many (identical) diseases in common. HeteSim will perform poorly when drugs do not treat identical diseases. In contrast, AnnSim also considers diseases that are not identical but are similar based on the topology of the NCIt annotations. Finally, (1 − *d*_tax_) and (1 − *d*_ps_) only consider the topology of the drug terms in the NCIt and will ignore the annotation evidence.

First, we retrieved from the LinkedCT dataset (LinkedCT.org, November 2011) interventions (diseases) associated with these drugs and consider as annotations the corresponding set of terms in the NCIt, i.e. each drug is annotated with the set of NCIt terms that correspond to the interventions related to these drugs in LinkedCT; the cardinality of these sets varies from 1 to 136. Table 10 reports on the values of these four similarity measures when Alemtuzumab is compared with the 11 other drugs in the dataset. We can observe that HeteSim consistently assigns very low values of similarity. Although all these drugs are used to treat different types of cancers, Alemtuzumab shares only a small number of identical diseases with the rest of the 11 drugs and this confuses HeteSim. AnnSim, however, assigns higher values because is able to detect that many of the diseases treated with Alemtuzumab share similar topological properties in NCIt with the diseases treated by the rest of the drugs. What is notable is that the taxonomic measures (1 − *d*_tax_) and (1 − *d*_ps_) only consider the topology of the drug terms in the NCIt and they ignore the annotation evidence. Thus, they return uniformly high similarity scores. The column ‘Annotation Count’ of Table 11 summarizes the number of annotations for each drug; it is clear that there is a wide variation in the diseases that are treated by these drugs. Hence, the inability to exploit the annotation evidence does not allow the taxonomic measures to differentiate between these drugs.

**Table 10.** Pairwise comparison of alemtuzumab with the rest of the 11 drugs

Pair drug	AnnSim	1 – *d*_tax_	1 – *d*_ps_	HeteSim
Alemtuzumab - Bevacizumab	0.263	0.670	0.500	0.001
Alemtuzumab - Brentuximab vedotin	0.140	0.364	0.222	0.000
Alemtuzumab - Catumaxomab	0.199	0.364	0.222	0.000
Alemtuzumab - Cetuximab	0.359	0.727	0.571	0.000
Alemtuzumab - Edrecolomab	0.037	0.727	0.571	0.000
Alemtuzumab - Gemtuzumab	0.046	0.500	0.333	0.000
Alemtuzumab - Ipilimumab	0.482	0.727	0.571	0.005
Alemtuzumab - Ofatumumab	0.468	0.727	0.571	0.002
Alemtuzumab - Panitumumab	0.422	0.727	0.571	0.000
Alemtuzumab - Rituximab	0.409	0.727	0.571	0.002
Alemtuzumab - Trastuzumab	0.319	0.727	0.571	0.000
**Average**	**0.286**	**0.635**	**0.479**	**0.001**

HeteSim assumes perfect matching between annotations and assigns low similarity values.

**Table 11.** Identifiers of the 12 anticancer drugs in the intersection of monoclonal antibodies and antineoplastic agents

ID	Drug	Annotation count
1	Alemtuzumab	39
2	Bevacizumab	136
3	Brentuximab vedotin	3
4	Catumaxomab	7
5	Cetuximab	50
6	Edrecolomab	1
7	Gemtuzumab	1
8	Ipilimumab	22
9	Ofatumumab	18
10	Panitumumab	22
11	Rituximab	100
12	Trastuzumab	18

Table 12 summarizes the pairwise scores for the four measures for each drug, compared with the other 11 drugs. For each drug, the score is used to rank the other 11 drugs. Finally, Table 13 presents SRank_1_ and SRank_2_. SRank_1_ is the Spearman’s correlation for AnnSim and (1 − *d*_tax_) and SRank_2_ is the correlation for AnnSim and (1 − *d*_ps_). We observe that HeteSim consistently assigns very low values of similarity. AnnSim again assigns higher values overall, this may be caused by the large variability of annotations of these drugs, i.e. the cardinality of the annotations considerably differs, and the pairwise intersection of the annotations is small. Values of SRank_1_ and SRank_2_ are higher than 0.5, suggesting that the annotation evidence is consistent with the topological relationships of the drugs in the NCIt.

**Table 12.** Average similarity and standard deviation (avg; std) when each is compared with 11 other drugs (antineoplastic agents and monoclonal antibodies)

ID	AnnSim	(1 – *d*_tax_)	(1 – *d*_ps_)	HeteSim
1	(0.286; 0.161)	(0.635; 0.150)	(0.479; 0.146)	(0.001; 0.002)
2	(0.206; 0.173)	(0.636; 0.152)	(0.479; 0.146)	(0.002; 0.002)
3	(0.206; 0.125)	(0.433; 0.093)	(0.284; 0.091)	(0.002; 0.007)
4	(0.244; 0.106)	(0.416; 0.066)	(0.269; 0.061)	(0.002; 0.003)
5	(0.303; 0.189)	(0.691; 0.163)	(0.547; 0.171)	(0.003; 0.004)
6	(0.157; 0.211)	(0.691; 0.162)	(0.547; 0.171)	(0.004; 0.014)
7	(0.157; 0.219)	(0.539; 0.045)	(0.375; 0.046)	(0.000 0.000)
8	(0.363; 0.208)	(0.691; 0.163)	(0.547; 0.171)	(0.004; 0.003)
9	(0.302; 0.159)	(0.692; 0.162)	(0.547; 0.171)	(0.003; 0.007)
10	(0.358; 0.212)	(0.692; 0.162)	(0.547; 0.171)	(0.007; 0.014)
11	(0.222; 0.169)	(0.691; 0.163)	(0.547; 0.171)	(0.001; 0.001)
12	(0.304; 0.175)	(0.692; 0.162)	(0.547; 0.171)	(0.002; 0.003)
Average	(0.259; 0.176)	(0.625; 0.137)	(0.476; 0.141)	(0.003; 0.005)

IDs are presented in Table 11.

**Table 13.** Spearman’s correlation for AnnSim and (1 − *d*_tax_) (SRank_1_) and the correlation for AnnSim and (1 − *d*_ps_) (SRank_2_)

ID	SRank_1_	SRank_2_
1	0.625	0.625
2	0.505	0.543
3	0.752	0.752
4	0.348	0.339
5	0.523	0.507
6	−0.318	−0.318
7	0.511	0.466
8	0.502	0.502
9	0.382	0.411
10	0.514	0.525
11	0.311	0.311
12	0.350	0.364
**Average**	**0.417**	**0.419**

IDs are presented by Table 11.

We note on a couple of outlier cases. Both Edrecolomab and Gemtuzumab have a single annotation, Colorectal Carcinoma and Acute Myeloid Leukemia, respectively. Although these diseases are different, the drugs have very similar and low values for AnnSim. We note that the drugs have high values for the taxonomic measures; e.g. [1 − *d*_tax_(Colorectal Carcinoma, Acute Myeloid Leukemia)] is equal to 0.714. Since *d*_tax_ meets the triangle inequality property (7), any disease that is similar to one disease will also be similar to the other. We further note that the SRank_1_ and SRank_2_ have a negative score for Edrecolomab but the score is closer to 0.5 for Gemtuzumab. This reflects that further work is needed to tune these measures to consider outliers.

Additionally, 7 of these 12 drugs were associated with diseases from the DrugBank SPARQL endpoint (http://wifo5-03.informatik.uni-mannheim.de/drugbank/snorql/), and the corresponding NCIt terms of these diseases were considered as the annotations of these drugs. These sets are smaller, share annotations and are more uniform in terms of size, i.e. the cardinality varies from 4 to 14. Table 14 summarizes the pairwise scores for AnnSim for each of the seven drugs, compared with the other six drugs. We can observe that given the homogeneity of these annotations, AnnSim is able to assign higher values of similarity. These results suggest that annotations impact on the values of similarity. Nevertheless, the effects may vary considerably depending on the characteristics of the annotations.

**Table 14.** Average similarity and standard deviation (avg; std) of AnnSim for 7 out of the 12 anticancer drugs in the intersection of monoclonal antibodies and antineoplastic agents

ID	Drug	AnnSim values
1	Alemtuzumab	(0.757; 0.315)
2	Bevacizumab	(0.702; 0.285)
5	Cetuximab	(0.738; 0.143)
7	Gemtuzumab	(0.757; 0.316)
10	Panitumumab	(0.254;0.130)
11	Rituximab	(0.757; 0.315)
12	Trastuzumab	(0.636; 0.156)
**Average**		**(0.661; 0.243)**

Annotations correspond to NCIt terms of the diseases associated with these drugs at the DrugBank SPARQL endpoint.

Details of drugs in Dataset 2 as well as their annotations and pairwise values of AnnSim can be found at http://pang.umiacs.umd.edu/AEDdemo.html.

### Effectiveness in dataset 3

The goal of this experiment is to analyze the correlation of AnnSim with respect to three standards of evaluation: EC*,* Pfam and SeqSim. First, we compute AnnSim for the pairs of proteins in Dataset 3 and then, we use the online tool Collaborative Evaluation of GO-based Semantic Similarity Measures (CESSM) to determine the correlation of AnnSim to the three standards of evaluation and to the semantic similarity measures presented in Table 3. These similarity measures extend Resnik’s (29), Lin’s (15) and Jiang and Conrath’s (18) measures to consider GO annotations of the compared proteins and the IC of these annotations; i.e. they use more domain knowledge (features) than AnnSim. Additionally, pairwise combinations of the annotations and their common ancestors are considered. The average combination which is labeled A, considers the average of the ICs of pairs of common ancestors. Campo *et al*. (42) applies the corresponding measure, i.e. the Resnik’s (29), Lin’s (15) and Jiang and Conrath’s (18) measures, to the maximum value of IC of pairs of common ancestors; these combined measures are distinguished with the labeled M. Further, Couto *et al*. (43) propose a measure which only the best-match average of the ICs of pairs of disjunctive common ancestors (DCA); the new measures are labeled B. Finally, the set-based measures simUI and simGIC (17) that extend the Jaccard index are also considered in the study.

**Figure 4.**
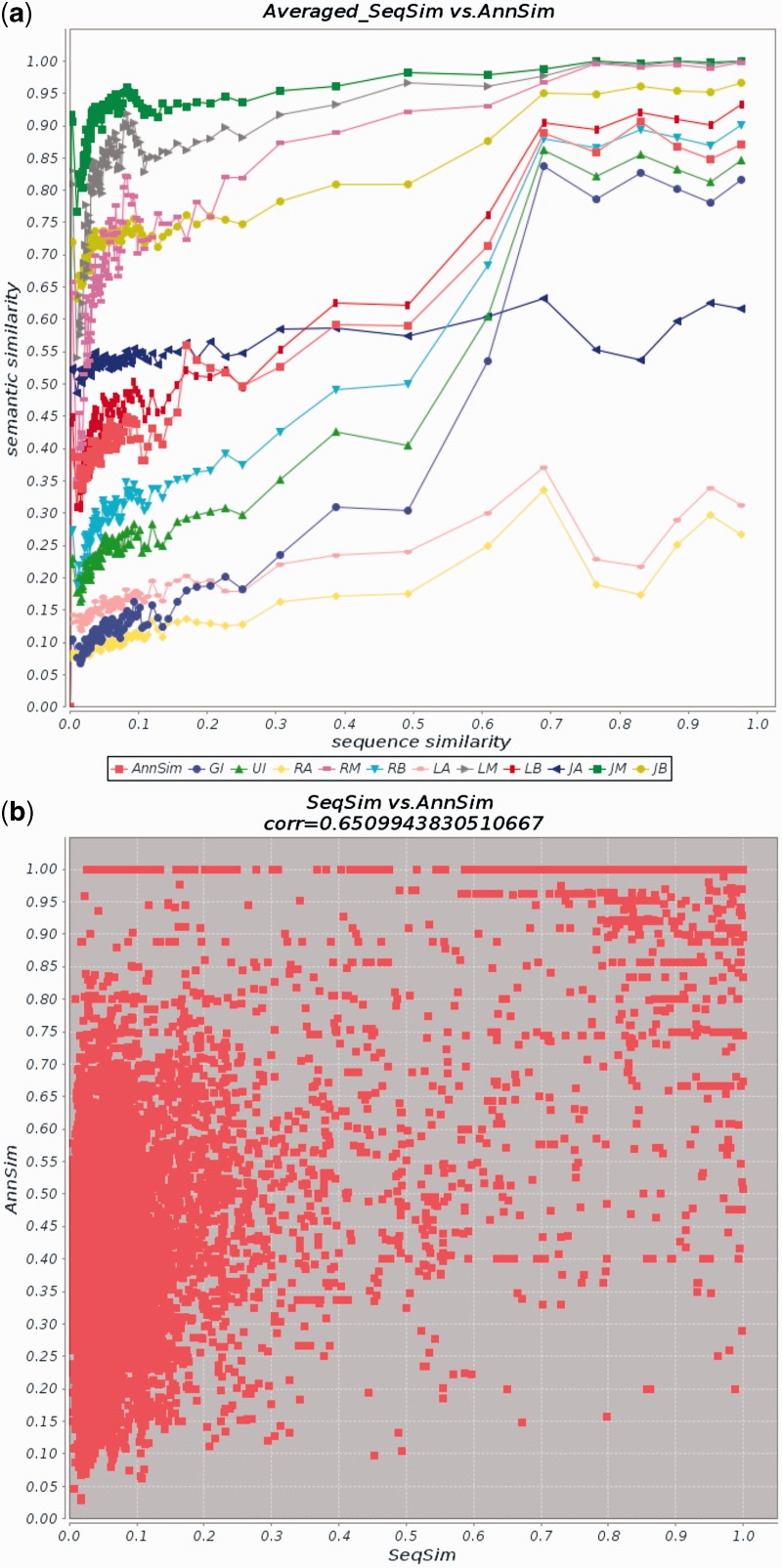
Comparison of AnnSim with SeqSim and similarity measures from Table 3. Results are produced by CESSM for GO BP terms. (**a**) Average values for AnnSim, the measures in Table 3 and SeqSim. (**b**) Plot of AnnSim and SeqSim scores (Pearson’s correlation of 0.65). The similarity measures are simUI (UI), simGIC (GI), Resnik’s Average (RA), Resnik’s Maximum (RM), Resnik’s Best-Match Average (RB), Lin’s Average (LA), Lin’s Maximum (LM), Lin’s Best-Match Average (LB), Jiang and Conrath’s Average (JA), Jiang and Conrath’s Maximum (JM), Jiang and Conrath’s Best-Match Average (JB).

Figure 4a and b reports the results of the comparison restricted to the GO BP terms. Figure 4a compares AnnSim with the GO-based extensions of the Resnik’s (29), Lin’s (15) and Jiang and Conrath’s (18) measures. Table 15 presents the Pearson’s correlation of AnnSim and the 11 semantic similarity measures presented in Table 3. Correlations of the column SeqSim on the Table 15 correspond to the Figure 4a. We observe that AnnSim provides the highest correlation coefficient with respect to Pfam. Furthermore, the correlation coefficient between SeqSim and AnnSim is the fourth highest and between EC and AnnSim is the fifth highest. AnnSim is more correlated to SeqSim, EC and Pfam than all the extensions of the Jiang and Conrath’s measure (18). Nevertheless, simGIC, simUI and RB exhibit better performance than AnnSim with respect to SeqSim and EC similarities. LB has higher correlation than AnnSim with EC similarity. Similar to AnnSim, these measures consider the GO annotations of the proteins. However, they additionally exploit information context of the GO annotations in conjunction with the most informative ancestors of these annotations; thus, a more precise estimate of the relatedness of two proteins is computed. Table 15 presents the *P* values for the correlation coefficients of AnnSim considering the null hypothesis that AnnSim coefficient is equal to the coefficients of the similarity measures presented in Table 3. To compute the *P* value, we used the Fisher’s *z* transformation and a one-sample *z* test for a correlation coefficient, described in (51) (eqs. 11.21 and 11.22). Fisher’s statistics has been used in previous semantic similarity studies (19, 51). AnnSim presents a statistically significant increase of the correlation coefficients (*P* value < 0.01) for all correlation coefficients except for four. AnnSim obtained low statistical significance increase for the correlations of GI and UI with respect to EC and Pfam, because the correlations of AnnSim, GI and UI are similar in for EC and Pfam.

**Table 15.** Pearson’s correlation coefficient between the three standards of evaluation and the 12 similarity measures on dataset 3

Similarity measure	SeqSim	*P*	EC	*P*	Pfam	*P*
GI	**0.7733**	<0.01	0.3981	0.4468	0.4547	0.1593
UI	0.7304	<0.01	0.4023	0.1810	0.4505	0.0440
RA	0.4068	<0.01	0.3022	< 0.01	0.3232	<0.01
RM	0.3027	<0.01	0.3076	<0.01	0.2627	<0.01
RB	0.7397	<0.01	**0.4444**	<0.01	0.4588	<0.01
LA	0.3407	<0.01	0.3041	<0.01	0.2866	<0.01
LM	0.2540	<0.01	0.3134	<0.01	0.2064	<0.01
LB	0.6369	< 0.01	0.4352	<0.01	0.3727	<0.01
JA	0.2164	<0.01	0.1931	<0.01	0.1732	<0.01
JM	0.2350	<0.01	0.2541	<0.01	0.1649	<0.01
JB	0.5864	<0.01	0.3707	<0.01	0.3319	<0.01
AnnSim	0.6510	–	0.3926	–	**0.4643**	–

The *P* values represent the probability of obtaining the correlation coefficient for AnnSim, EC and Pfam assuming the correlation coefficient of other 11 similarity measures. The higher correlation in each standard of evaluation is highlighted in bold.

Figure 4b reports on the Pearson’s correlation between AnnSim and SeqSim of 0.65; this indicates a moderately strong relationship. These results suggest that, as expected, the domain-specific measures that use additional knowledge exhibit the best performance. However, the behavior of AnnSim reflects that even it is a general measure, it is able to successfully exploit knowledge encoded in the protein annotations.

### Effectiveness in datasets 4 and 5

The goal of this experiment is to evaluate the quality of AnnSim with respect to domain-specific similarity measures. We consider Datasets 4 and 5 that contain drug and target interactions and evaluate the quality of AnnSim and domain-specific measures in terms of the quality assessed by the state-of-the-art clustering techniques when these measures are used. Diverse clustering algorithms provided by the WEKA (http://www.cs.waikato.ac.nz/ml/weka/) tool are used in the evaluation. Furthermore, we built our gold standard clustering by grouping together in a cluster only drugs that share exactly the same set of categories, i.e. the average Category-based Score of our gold standard clustering is 1.0. Information about the category of the drugs was downloaded from the DrugBank website (http://www.drugbank.ca/ February 2014).

First, for drug–target interactions in Dataset 4, we compare the quality of AnnSim and five drug–drug similarity measures in Table 5 in terms of similarity of clusterings produced using AnnSim and these measures. Clustering similarity is computed with two different measures: average Category-based Score (C) and Jaccard Clustering Index (J) (52).

Given a clustering *C* of drugs, the average Category-based Score, C(*C*), corresponds to the average of the ‘Category-based’ measure for each pair of drugs in the clusters of *C*. Values of C(*C*) ranges between 0.0 and 1.0. A value equal to 0.0 indicates that there is no intersection between the categories of the pairs of drugs in the clusters of *C*, whereas a value closed to 1.0 represents that almost all the pairs of drugs in each cluster of *C* share exactly the same categories. Table 16 illustrates the results of computing the average Category-based Score measure on the clusterings produced by the Expectation Maximization (EM) clustering algorithm (53) of WEKA. We ran EM for each of the five drug–drug similarity measures and for three versions of AnnSim, i.e. one version per target–target similarity measure ‘seq’, ‘dist’ and ‘go’. EM was run for 10M of iterations until 259 clusters were produced. We can observe that all the clusterings are characterized by high values of the average Category-based Score. These high values indicate that both similarity measures and the EM clustering algorithm are able to placed together in a cluster drugs that share the majority of their categories. Particularly, we can highlight the average Category-based Score value of the clustering of **ATC** and the values of **AnnSim_seq_**, **AnnSim_dist_** and **AnnSim_go_-**based clusterings. First, both **ATC** and AnnSim rely on annotations to measure the relatedness of drugs. Because **ATC** is a domain-specific measure, it is able to better capture the particular properties of the drugs. Nevertheless, although AnnSim is a general-purpose measure, it can exhibit good quality independently of the target–target measure used to compute the similarity of the targets that annotate the drugs. This result supports the assumption that AnnSim is stable even if properties of the drugs change.

**Table 16.** Average similarity of the 259 clusters of the clustering obtained using the an EM algorithm for each drug–drug measure on 310 drugs

AnnSim_seq_	AnnSim_dist_	AnnSim_go_	ATC	Chem.	Ligand	CMap	SideEff.
0.8939	0.8939	0.8939	0.9129	0.8737	0.8727	0.8304	0.8746

**Figure 5.**
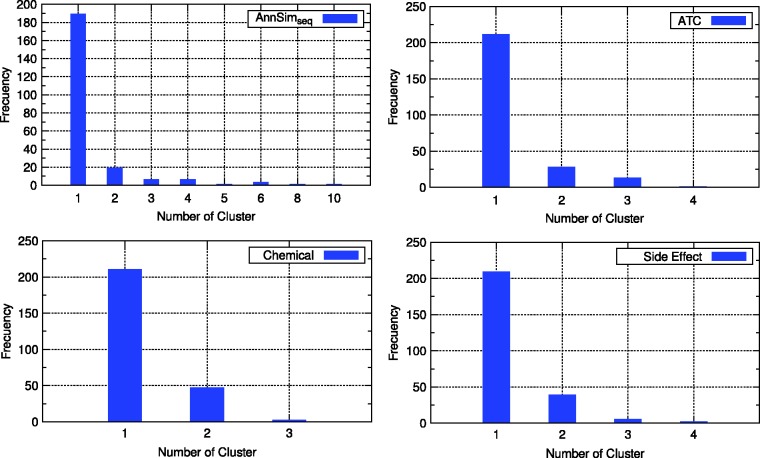
Distribution of the number of clusters of the clustering obtained by four drug–drug similarity measure.

It is important to notice that clusterings of **ATC**, **Chem.**, **Ligand**, **CMap** and **SideEff.** comprise a large number of clusters with one drug, whereas the three versions of AnnSim produce larger-sized clusters. Figure 5a–d presents the distribution of the number of clusters with a given number of drugs for clustering of **AnnSim_seq_**, **ATC**, **Chem.** and **SideEff.**, respectively.

Although **AnnSim_seq_** clustering is composed of larger-sized clusters, e.g. clusters with 6, 8 and 10 drugs, the drugs on these clusters share a high number of categories. Table 17 presents the drug frequency per category in each cluster, for clusters with 10 and 6 drugs. We can observe that the majority of the drugs in each of these clusters, share at least one category. Additionally, drugs in the clusters related with the categories highlighted in **bold**, share at least on target. For example, drugs in the cluster with 10 elements that are associated with the categories ”Anti-inflammatory Agents, Steroidal”, Anti-inflammatory and Anti-inflammatory Agents, are all related to the target Glucocorticoid receptor. Also, drugs associated with the category Glucocorticoids are related to the same target. This pattern suggests that drugs in the same cluster sharing at least one category, also share at least one target. This pattern may correspond to a potential association discovered by AnnSim that could be not observed in the other clusterings.

**Table 17.** Description of three clusters obtained using AnnSim measure and the EM clustering algorithm of WEKA

No. of elements in the cluster	DrugBank drug categories In the cluster	No. of drugs with this category
10	Immunosuppressive agents	1
	Neuroprotective agents	1
	**Anti-inflammatory agents**	**10**
	Antipruritic agents	1
	**Corticosteroid**	**2**
	Antiemetics	1
	Anti-asthmatic agents	1
	**Anti-INFLAMMATORY**	1
	**“Anti-inflammatory agents, steroidal**	1
	Anti-allergic agents	1
	Steroidal	1
	**Corticosteroids**	**2**
	**Glucocorticoids**	**8**
	Adrenergic agents	3
	Antineoplastic agents	1
	“Antineoplastic agents	1
	**“Corticosteroids**	**1**
6	Sympathomimetic	1
	Anti-anxiety agents	1
	Vasodilator agents	1
	**Adrenergic beta-antagonists**	**5**
	Sympathomimetics	1
	Anti-arrhythmia agents	**4**
	Cardiotonic agents	1
	EENT drugs	1
	**Adrenergic beta-agonists**	**1**
	Sympatholytics	3
	Antihypertensive agents	4
6	Nucleic acid synthesis inhibitors	3
	“Antibiotics	1
	Anti-bacterial agents	1
	Enzyme inhibitors	1
	**Anti-infectives**	**2**
	Photosensitizing agents	1
	Antibiotics	1
	**Anti-infective agents**	**3**
	Analgesics	1
	Quinolones	2
	**“Anti-infective agents**	**1**
	Antitubercular agents	1
	Antineoplastic agents	2

One cluster with 10 elements and two with six elements are shown. We highlight in bold similar category terms or terms with high frequency. Cluster with nine elements, their targets and frequency of interactions.

**Figure 6.**
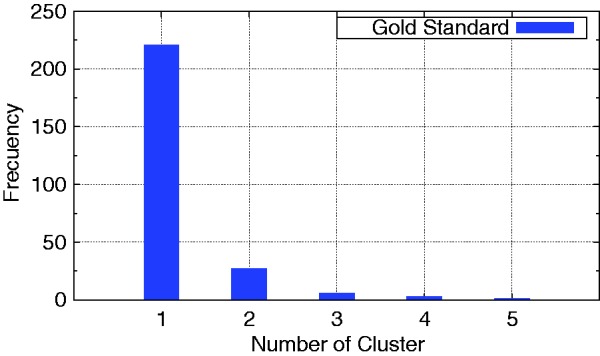
Distribution of the number of cluster of our gold standard clustering.

We also measure the quality of the clustering of these similarity measures, based on how similar these clusterings are to the ‘gold standard’ clustering. Figure 6 presents the drug frequency distribution of our gold standard clustering. As can be observed, our gold standard clustering is composed of clusters of up to five drugs and more than 200 clusters with only one drug.

We use the Jaccard Clustering index to compare all these clusterings of the drugs with respect to our gold standard clustering. Jaccard Clustering index (J) measures similarity of two clusterings in terms of the number of pairs that are in the same cluster in the clusterings. J(*C*1,*C*2) is computed as the number of pairs that are in the same cluster in *C*1 and *C*2 divided by, this number plus the number of pairs that are in one cluster in either *C*1 or *C*2, but not in both. Values of Jaccard index are in the range of [0.0;1.0]. J(*C*1,*C*2) is 1.0, if and only if, the pairs of drugs that appear together in a cluster in *C*1 are exactly the same to the pairs that appear in a cluster in *C*2, i.e. *C*1 and *C*2 have exactly the same clusters. On the other hand, J(*C*1,*C*2) is 0.0, if and only if, there is no pair of drugs that appear together in one cluster of *C*1 (respectively, *C*2) and appear together in a cluster of *C*2 (respectively, *C*1).

Table 18 reports on the Jaccard Clustering index of all the eight clusterings with respect to our gold standard clustering. We can observe that the versions of AnnSim have the lowest values of this measure. This behavior is caused by the distribution of clusters generated by AnnSim, where drugs that share one category instead of all their categories, are placed in the same cluster. Although this may look a disadvantage of AnnSim, we consider that grouping terms that share at least one property can be useful in data mining process where the discovery of properties of similar but no equal objects, is an important task. For example, we could suggest a potential relationship between Anti-inflammatory Agents drugs and Glucocorticoids drugs, as well as, between their targets.

**Table 18.** Jaccard similarity coefficient between each drug–drug measure clustering and the ground truth clustering

AnnSim_seq_	AnnSim_dist_	AnnSim_go_	ATC	Chem.	Ligand	CMap	SideEff.
0.5657	0.5657	0.5657	0.7175	0.7512	0.7431	0.7045	0.7211

Similarly, for drug–target interactions in Dataset 5, we compute clusterings of the drugs that comprise the four sub-sets of the dataset. Clusterings are computed for both AnnSim and the drug–drug measure computed by SIMCOMP (‘Sim’). Because there are drugs in Dataset 5 that are not associated with a category in DrugBank, we could not build the baseline partition as in the previous experiment. Alternatively, we evaluate the quality of the clusterings based on intra- and inter-similarity measures that indicate how similar are the drugs placed in one clusters and how distant are the centroids of the clusters that comprised the clusterings, respectively. The center-based algorithm *k-means* provided by WEKA is used to compute the clusters with an input of 259 centers. We compute two clustering similarity measures: the Davies–Bouldin index (54) and the ‘Coupling’ measure (55).

The Davies–Bouldin index (54) relies on the values of a radio of intra-cluster and between-cluster distances. Given a clustering of *k* clusters, the Davies–Bouldin index is defined as follows:

$$\frac{1}{k}\sum_{i=1}^{k}\left({\text{max}}_{i\neq j\{D_{i,j}\}}\right)$$

where, *D_i_*_,_*_j_* is the intra-to-between cluster distance ratio for the *i*th and *j*th cluster, i.e. $D_{i,j}=\frac{d_{i}+d_{j}}{d_{i,j}}$. A value of *d_i_* corresponds to the average distance between each point in the *i*th cluster and the centroid of the *i*th cluster. Although a value of *d_i_*_,_*_j_* is the average distance between each point in the *i*th cluster and the centroid of the *j*th cluster, we use the Euclidean distance to compute the distance between centroids and a point and a centroid. The maximum value of *D_i_*_,_*_j_* represents the worst-case intra-to-between cluster ratio for the *i*th cluster. Optimal clusterings are characterized by the smallest Davies–Bouldin index value.

On the other hand, the Coupling measure (55) indicates the similarity of the entities in two different clusters. Given a clustering of *k* clusters, the Coupling measure is defined as follows:

$$\frac{\sum_{i>j}\text{S}\text{im}(C_{i},C_{j})}{\frac{k(k-1)}{2}}$$

where, *C_i_* and *C_j_* are the centroids of the *i*th and *j*th clusters, respectively. We use the cosine similarity to compute Sim(*C_i_,C_j_*). Optimal clusterings are characterized by the lowest values of the Coupling measure, i.e. clusterings whose centroids are not similar. Table 19 illustrates the values of the intra-clustering similarity Davies–Bouldin index and the values of the inter-clustering Coupling measure. We can observe that for the Davies–Bouldin index and the Coupling measure, AnnSim and Sim have low values. Nevertheless, AnnSim slightly surpasses Sim in the two measures; these results suggest that AnnSim-based clustering is closer to the optimal clustering than the Sim-based clustering.

**Table 19.** Comparison of clusterings produced by *K* means with 259 centers for AnnSim and Sim (drug–drug similarity measure computed by SIMCOMP)

Enzyme		GPCR		ion		nr	
AnnSim	Sim	AnnSim	Sim	AnnSim	Sim	AnnSim	Sim
Davies–Bouldin index (54)							
1.27	1.97	**1.04**	**1.95**	1.12	1.63	**0.65**	**1.03**
Coupling measure (55)							
0.05	0.06	0.07	0.08	0.07	0.08	0.16	0.17

Davies–Bouldin index indicates how distant the points in a cluster are, i.e. low values suggest that drugs in a cluster are similar. The Coupling Measure indicates how similar centroids in a clustering are, i.e. low values suggest that the centroids are distant. More distant values are highlighted in bold**.**

Table 20 presents the targets associated with the drugs in one of the clusters in the clustering of the GPCRs using AnnSim; additionally, the number of interactions is reported. We can observe that in this cluster, 19 out of 27 interactions between the nine drugs and their targets correspond to interactions with a target of the class Gamma-aminobutyric-acid receptor. This result corroborates the pattern suggested in Dataset 4, where drugs placed in the same cluster are very likely to interact with the same targets. Because information about the similarity between the targets was not considered by AnnSim, these patterns could not be identified by clustering these drugs in terms of this measure. In terms of discovery, the identified patterns corroborate hypothesis of existing drug–target link prediction approaches (20), which state that similar drugs are related to similar targets. Thus, the clusterings obtained using AnnSim could be used as input of state-of-the-art link prediction approaches to support the prediction of potential new interactions between drugs and targets.

**Table 20.** Description of a cluster in the GPCR obtained using AnnSim measure

Target	No. of interactions
Androgen receptor	1
Gamma-aminobutyric-acid receptor class	19
Heat shock protein HSP 90-alpha	1
Mineralocorticoid receptor	1
16S rRNA	1
C-1-tetrahydrofolate synthase, cytoplasmic	1
Glucocorticoid receptor	1
Inosine-5′-monophosphate dehydrogenase 1	1
30S ribosomal protein S12	1

Cluster with nine elements, their targets and frequency of interactions.

## Conclusions and future work

We have proposed an annotation similarity measure called AnnSim to determine the relatedness of two entities based on the similarity of their sets of annotations. AnnSim is defined as a 1–1 maximum weight bipartite matching. We have performed an extensive evaluation using multiple datasets and ground truths. First, we evaluated the quality of existing taxonomic distances with respect to multiple ontologies, then these taxonomic measures and ontologies were used to compute AnnSim. The observed results corroborate that AnnSim is stable across different taxonomic measures and ontologies. Furthermore, we use the online tool CESSM for the automated evaluation of GO-based semantic similarity measures on GO terms, the sequence similarity and AnnSim. The observed results suggest that AnnSim can also be used to explore and explain deeper and more nuanced relationships among proteins or drug families. These relationships are moderately strong to strong correlated to domain-specific measures. Finally, AnnSim was compared with a great variety of domain-specific similarity measures to compute relatedness of drugs and targets. An extensive evaluation was conducted on the quality of the clusterings obtained from these measures. We could observe that although AnnSim is a general-purpose measure that does not exploit knowledge or properties of a particular domain, it is competitive with a variety of domain-specific measures. The reported results can be used to suggest or discover potential relationships between scientific entities. Although AnnSim exhibits a good behavior in a diversity of datasets, we note that the 1–1 maximum weight bipartite matching has many limitations since it ignores unmatched terms and does not consider groups of matching terms. In future work, we will explore extensions to ‘*n*–*m* maximum weight bipartite matching’ to uncover potential relationships between terms that may contribute to more precisely measurements of relatedness between scientific entities and to suggest potential novel patterns.

## Funding

This research has been partially funded by National Science Foundation (NSF) grant 1147144 and DID-USB.

*Conflict of interest:* None declared.
